# Genome-wide association study reveals serovar-associated genetic loci in *Riemerella anatipestifer*

**DOI:** 10.1186/s12864-024-09988-4

**Published:** 2024-01-13

**Authors:** Zhishuang Yang, Xueqin Yang, Mingshu Wang, Renyong Jia, Shun Chen, Mafeng Liu, Xinxin Zhao, Qiao Yang, Ying Wu, Shaqiu Zhang, Juan Huang, Xumin Ou, Sai Mao, Qun Gao, Di Sun, Bin Tian, Dekang Zhu, Anchun Cheng

**Affiliations:** 1 Research Center of Avian Diseases, College of Veterinary Medicine, Sichuan Agricultural University, Chengdu, Sichuan China; 2 Key Laboratory of Animal Disease and Human Health of Sichuan Province, Chengdu, Sichuan China; 3 International Joint Research Center for Animal Disease Prevention and Control of Sichuan Province, Chengdu, Sichuan China; 4 Engineering Research Center of Southwest Animal Disease Prevention and Control Technology, Ministry of Education of the People’s Republic of China, Chengdu, Sichuan China

**Keywords:** *Riemerella anatipestifer*, Pan-genome wide association studies, Capsule, Serovar-associated

## Abstract

**Background:**

The disease caused by *Riemerella anatipestifer* (*R. anatipestifer*, RA) results in large economic losses to the global duck industry every year. Serovar-related genomic variation, such as the O-antigen and capsular polysaccharide (CPS) gene clusters, has been widely used for serotyping in many gram-negative bacteria. RA has been classified into at least 21 serovars based on slide agglutination, but the molecular basis of serotyping is unknown. In this study, we performed a pan-genome-wide association study (Pan-GWAS) to identify the genetic loci associated with RA serovars.

**Results:**

The results revealed a significant association between the putative CPS synthesis gene locus and the serological phenotype. Further characterization of the CPS gene clusters in 11 representative serovar strains indicated that they were highly diverse and serovar-specific. The CPS gene cluster contained the key genes *wzx* and *wzy*, which are involved in the Wzx/Wzy-dependent pathway of CPS synthesis. Similar CPS loci have been found in some other species within the family *Weeksellaceae*. We have also shown that deletion of the *wzy* gene in RA results in capsular defects and cross-agglutination.

**Conclusions:**

This study indicates that the CPS synthesis gene cluster of *R. anatipestifer* is a serotype-specific genetic locus. Importantly, our finding provides a new perspective for the systematic analysis of the genetic basis of the *R anatipestifer* serovars and a potential target for establishing a complete molecular serotyping scheme.

**Supplementary Information:**

The online version contains supplementary material available at 10.1186/s12864-024-09988-4.

## Background

*Riemerella anatipestifer* (*R. anatipestifer*, RA) is an important veterinary pathogen belonging to the family *Weeksellaceae* [1]. RA poses a threat to domestic ducks, geese, and turkeys, causing acute or chronic septicemia [2]. Since 1982, when Bisgaard established the *R. anatipestifer* serotyping scheme [3], at least 21 serovars have been reported around the world [4]. And there was no cross-reactivity between different serovars [5]. There are several sets of reference strains that have been used for serovar designation by different groups [3, 6, 7]. Recently, Omaleki et al. reconfirmed the serovar reference strains and identified 17 different serovars, which is the latest validated serotyping scheme [8]. Nonetheless, the serotyping of *R. anatipestifer* still relies heavily on reference strains and antisera, and no molecular serotyping methods have been proposed.

In most bacteria, the surface polysaccharide structures exhibit intraspecies diversity, which is usually associated with serological phenotype [9, 10]. The capsular polysaccharide (CPS) synthesis gene cluster was frequently utilized as a molecular serotyping target due to its genetic diversity [9, 11, 12]. Notably, due to the high correlation between the genetic signature of CPS gene cluster and the serovar phenotype, CPS related genes have been widely used as targets for molecular serotyping of many bacteria [12–14]. The most commonly targeted genes are *wzx* and *wzy*, which encode the oligosaccharide unit flippase (Wzx) and polymerase (Wzy), respectively. This gene pair plays a crucial role in CPS synthesis, which is directly linked to the specificity of CPS [11, 13]. Several studies have focused on the genes that are related to lipopolysaccharide (LPS) and CPS in *R. anatipestifer* [15–17], but the information they provide regarding serovar characteristics is limited. Previous studies have speculated that the serological characteristics of *R. anatipestifer* are associated with surface polysaccharides [18]. Our recent study indicates that the CPS of *R. anatipestifer* determines the serological specificity of serovar 2, rather than the LPS [19], while the genetic loci determining the serovars of *R. anatipestifer* remain unclear.

With the development of whole-genome sequencing technology and the accumulation of genomic data, genome-wide association studies (GWAS) have become a powerful tool in bacterial research to reveal the genetic basis of important phenotypes. Recently, several methods have been introduced to assess the correlation between bacterial phenotypes and genotypes [20, 21]. Among these, the pan-genome-wide association study (Pan-GWAS), based on the typical gene presence/absence features of bacterial accessory genomes, has proven effective in studies of bacterial resistance and pathogenicity [22, 23]. There have been several pan-genomic studies of *R. anatipestifer* [24, 25]. However, to the best of our knowledge, no study has used the pan-GWAS approach to determine the association between genotypes and phenotypes in *R. anatipestifer*.

In the current study, we used pan-GWAS to identify the genetic loci associated with serovars. And we further analyzed the biological functions of the serologically associated genetic loci and preliminarily characterized our findings using gene knockout methods. These findings will provide a basis for further exploration of the molecular mechanism of *R. anatipestifer* serological phenotypes and provide direction for the establishment of molecular serotyping methods.

## Materials and methods

### Bacterial strain and whole-genome sequencing

The *R. anatipestifer* strains and the published genome data employed in this study are listed in Supplementary Table 1. One representative strain of each serovar was chosen for further presentation. Serovar representative strains and serotyping information were shown in Table 1. And all representative strains were obtained from the Culture Collection University of Gothenburg (CCUG). Other strains of *R. anatipestifer* used in this study were identified, characterized and archived by the Research Center of Avian Diseases, Sichuan Agricultural University (Chengdu, Sichuan, China).

**Table 1:** Serovar representative strains in this study

CCUG ID of representative strains	Strains name	Serovar	Bisgaard (1982) Serovar Reference Strains [3]	Bisgaard (1982) Serovar [3]	Rubbenstroth et al. (2013) Reference strains [7]	Rubbenstroth et al. (2013) Serovar [7]	Omaleki et al. (2021) Bisgaard Serovar Reference Strains [8]	Omaleki et al. (2021) Bisgaard Serovar [8]
CCUG18373	P-892	U1			DRL-24105	1		
CCUG25001	HPRS 2591	2	HPRS 2591	2	DRL-24046	2	HPRS 2591	2
CCUG25002	HPRS 2212	3	HPRS 2212	3	DRL-26338	3	HPRS 2212	3
CCUG25004	HPRS 2514	5	HPRS 2514	5	DRL-24123	5	HPRS 2514	5
CCUG25005	HPRS 2336	6	HPRS 2336	6	P-2123	6	HPRS 2336	6
CCUG25008	HPRS 2174	8	HPRS 2174	8	HPRS-2199	10	HPRS 2174	8
CCUG25010	HPRS 2528	9	HPRS 2528	9	DRL-27179	7	HPRS 2528	9
CCUG25011	HPRS 2564	10	HPRS 2564	10	HPRS-2565	4	HPRS 2564	10
CCUG25013	Singapore 8	U2			DRL-28020	11		
CCUG25054	HPRS 2560	11	HPRS 2560	11	DRL-26220	8	HPRS 2560	11
CCUG25055	8755/9	12	8755/9	12	8755/9	12	8755/9	12

*CCUG *Culture Collection University of Gothenburg, *U1 *Undefined type 1, *U2 *Undefined type 2

The *R. anatipestifer* strains were grown in tryptic soy broth (TSB) and tryptic soy agar (TSA), at 37 °C for 12 h under microaerophilic conditions. *R. anatipestifer* genomic DNA was extracted using the TIANamp Genomic DNA Kit (TIANGEN BIOTECH, China). Whole-genome sequencing was performed using the Illumina HiSeq 2500 platform at the Beijing Genomics Institute (BGI, Shenzhen, China). Short-reads were filtered by Fastp (v0.19.4, default settings) [26] and draft genomes were assembled using SPAdes (v 3.11.0, default parameters with --careful flag) [27].

All *R. anatipestifer* strains were confirmed by RA-specific, 16S rRNA PCR assays (universal primer pairs 27F and 1410R), and genomic average nucleotide identity, as described previously [7, 28, 29]. and non-redundant isolates (> 2000 SNPs/INDELs) were identified using custom scripts based on the NUCmer program (version 4.0.0beta2, https://github.com/youngDouble/MUMmerSNP2VCF_script) [30].

### Agglutination test using the antisera

The serovars of *R. anatipestifer* involved in this study were determined by slide agglutination according to Bisgaard [3]. Standard serotyping antisera were obtained from RIPAC-LABOR GmbH (Potsdam, Germany), and those antisera have been extensively tested to indicate no cross-reactivity [6, 7]. As our representative strains are mainly from Bisgaard’s scheme [3], we have labelled the serovar types according to the recent study by Omaleki et al. [8]. For non-serovar reference strains, we have labelled them with temporary serovar types. The serovar represented by CCUG 18373 is labeled as undefined type 1 (U1) and the serovar represented by CCUG 25013 is labeled as undefined type 2 (U2) (Table 1).

### Genome wide association study of *R anatipestifer* serovar phenotypes

To explore the association between *R. anatipestifer* serovars and genetic characteristics, a pan-genome-wide association study (Pan-GWAS) was performed. To ensure statistical power, GWAS was performed for the three most prevalent serovars in China. Specifically, the *R. anatipestifer* genome was annotated using Prokka (version 1.14.6, default parameters) [31], and the pan-genome containing 45 strains of *R. anatipestifer* was reconstructed with Roary (version 3.12.0, with identity threshold of protein = 90) [32]. Furthermore, Scoary (v1.6.16) [20] was used to perform the Pan-GWAS with the *gene_presence_absence* file generated by Roary (only serovars containing more than 10 strains were considered). Scoary’s *P*-value and *Q*-value (adjusted *P*-value, adjust algorithm: Benjamini-Hochberg method) cut-offs were set to 0.05, the sensitivity cut-off was set to 95% and specificity to 85%. Next, we mapped the genes that were significantly associated with the serovar to the corresponding genome to obtain the distribution characteristics. Contig comparisons were generated with Easyfig (v2.2) [33].

### Functional speculation of the gene cluster

To explore the function of serovar-related genetic loci, genome-wide biosynthetic gene clusters (BGCs) of *R. anatipestifer* was predicted with antiSMASH (version 4.2.0, parameter setting: --clusterblast --subclusterblast --knownclusterblast --smcogs --inclusive --borderpredict) [34]. BGCs analysis was performed again by DeepBGC [35], which uses deep learning strategies to mine biosynthetic gene clusters in the microbial genome. The results of the above two methods will be considered comprehensive.

### Gene boundary determination of *R. anatipestifer* CPS gene cluster

Based on the results of biosynthetic gene cluster mining, we further determined the boundaries of the *R. anatipestifer* CPS gene cluster. Specifically, we retrieved 450 known CPS gene clusters from the NCBI Nucleotide database (https://www.ncbi.nlm.nih.gov/nuccore) (Supplementary Table 2). We downloaded the protein sequence of these gene clusters, used CD-HIT (version 4.8.1, parameter setting: -c 1 -aS 0.95) [36] to remove redundancies and constructed the database. TblastN (version 2.6+) was used to map these proteins to the *R. anatipestifer* genome, and the resulting filtering thresholds were as follows: coverage ≥50% (−qcov_hsp_perc 50), e-value≤1e-5 (−evalue 1e-5). Subsequently, the densely mapped regions in the genome are considered as candidates for the CPS gene cluster. Finally, combined with the prediction results of BGCs, the boundary of the gene cluster was determined by manual inspection.

### Annotation of the CPS synthesis gene cluster

Protein-encoding genes were predicted using Prokka [31] and NCBI Prokaryotic Genome Annotation Pipeline (PGAP) [37] with default parameters. To assign functions to the predicted genes, the Conserved Domains Database (CDD) [38] was used to search for conserved domains with an E-value threshold of 0.01. Meanwhile characteristic gene annotation of genes was performed using Blastp (v2.6+) against Non-Redundant (NR, https://ftp.ncbi.nlm.nih.gov/blast/db/) database. The E-value and query coverage were set at 1e-5 and 50% respectively. Wzx and Wzy are key proteins in CPS synthesis and possess a typical multi-transmembrane structure [39]. Therefore, for the identification of Wzx and Wzy, TMHMM2.0 [40] was used to predict the transmembrane regions of proteins.

### Inter- and intra-serovars comparison of CPS gene cluster

Gene cluster nucleotide sequence alignment was performed using MAFFT [41] in automatic mode, and then Mega X [42] with default parameters and 1000 bootstrap replicates were used to reconstruct the Neighbor-joining (NJ) [43] phylogenetic tree.

Blast (v2.6+) and Easyfig (v2.2) [33] were used for inter- and intra-serovar CPS gene cluster comparisons. Clustal Omega Web services (https://www.ebi.ac.uk/Tools/msa/clustalo/, default settings) was used to calculate the percentage identity of all Wzx and Wzy protein sequences.

### Conservation analysis of CPS locus in family *Weeksellaceae*

To investigate the conservation of the CPS locus of *R. anatipestifer* in closely related species within the family *Weeksellaceae*, the multi-gene search method was implemented against representative genome database (https://ftp.ncbi.nlm.nih.gov/genomes/refseq/, data as of November 12, 2022) [44]. Specifically, Multigeneblast [45] was used to find homologues of *R. anatipestifer* CPS gene cluster from the representative genomes of *Weeksellaceae* species (based on published data). In addition, we used Easyfig to compare the collinearity of the best homologues.

### Construction of *R. anatipestifer wzy* mutant strain CH-2Δ*wzy*

To further characterize the relationship between the predicted CPS gene cluster and capsule synthesis, we performed a deletion mutation in the predicted key gene. Briefly, the *wzy* gene (*G148_RS04365*) was deleted by allelic exchange using the recombinant suicide vector pYA4278 (Supplementary Fig. 1a, Kong et al. [46]; donated by Professor Kong). Briefly, upstream (L) and downstream (R) fragments of the *R. anatipestifer* CH-2 *wzy* gene were amplified by PCR from the genome using *wzy*-Left F and *wzy*-Left R, and *wzy*-Right F and *wzy*-Right R primers, respectively. A 1145-bp Spec^R^ cassette was PCR-amplified from the pYES1 new plasmid using the Spc F and Spc R primers. The three fragments were then spliced together in vitro by overlap extension using the *wzy*-Left F and *wzy*-Right R primers, producing the LSR fragment. Adenosine nucleotides were added to both ends of the PCR product, which was then ligated to the AhdI-digested T-cloning suicide vector pYA4278 to generate pYA4278-LSR, which carries a deletion of the entire *wzy* gene. Subsequently, pYA4278-LSR was successively transformed into *E. coli* X7213λpir [47]. *E. coli* X7213λpir (Donor) and *R. anatipestifer* CH-2 (Recipient) were mixed in a 10 mM MgSO_4_ solution and incubated on TSB agar with diaminopimelic acid at 37 °C for 24 h. Spec^R^ transconjugants were further selected in media containing spectinomycin (40 μg/ml). The detailed steps of this study refer to the methods of Luo et al. [28]. To confirm the *R. anatipestifer* mutant CH-2Δ*wzy*, we performed PCR targeting the transconjugants (see Supplementary Fig. 1b for details). The above strains and plasmids are preserved at the Research Center of Avian Diseases, College of Veterinary Medicine, Sichuan Agricultural University, Chengdu, Sichuan, China. The primers used for construction of the above strains and plasmids are listed in Table 2.

**Table 2:** Primers for identification of *wzy* deletion

Name of pimer	Targeted gene;Description	Product Length(bp)		Sequence	Source or reference
wzy-Left	G148_RS04360; Amplification of the *wzy* upstream fragment	609	F	AAGAACATTACCCATATCCTATCGTTTCGACGGTA	This study
			R	TTCTGTCCTGGCTGGTTTTACGAATATTTGTAAGATA	This study
wzy-Right	G148_RS04370; Amplification of the *wzy* downstream fragment	602	F	CCAAGGTAGTCGGCAAATAATTTTATGAAAAAAGTAC	This study
			R	TACATGAGAAACCACAAAAGCCTCTTTGGGAATA	This study
wzy	RA-CH-2 (G148_RS04365); Amplification of the wzy	886	F	TCCAATGGGTTTACTTTCTTGTAACTTTGTCT	This study
			R	CGTAATGGTTGGTTGAGATTCATTGGAG	This study
LSR	G148_RS04360 + spec; Identification of transconjugants	1199	F	AGGTAGATAGGGCAAGTATGGCTTTTTCG	This study
			R	ACCGTAACCAGCAAATCAATATCACTGTG	This study
16S rRNA_RA	16S rRNA; Identification of species	960	F	CTTCGGATACTTGAGAGCG	[28]
			R	GCAGCACCTTGAAAATTGT	[28]
Spec	*spec;* Amplification of the *spec*	1145	F	TCTTACAAATATTCGTAAAACCAGCCAGGACAGAAAT	This study
			R	ACTTTTTTCATAAAATTATTTGCCGACTACCTTGGTG	This study

### Microscopic imaging of capsules by India ink staining and transmission electron microscopy

Capsule staining of *R. anatipestifer* was carried out using an improved Indian ink staining method as previously described [48]. Briefly, one drop of Indian ink and one drop of bacterial suspension were mixed on a glass slide, spread thinly, and air-dried. The slide was then counterstained with 1% crystal violet for 1 minute, gently rinsed with distilled water, air-dried, and observed by optical microscope.

The wild-type (CH-2) and mutant (CH-2Δ*wzy*) strains were washed twice with phosphate-buffered saline at 5000 r/min for 10 minutes after overnight culture. The precipitate was treated with 2.5% glutaraldehyde (pH 7.2) for 2 hours. After washing 20 times with ultrapure water, the cells were adsorbed onto copper grids and then stained with the phosphotungstic acid solution for 5 minutes. The capsule was observed by field-emission transmission electron microscope (TEM, FEI Tecnai G2 F20, 200 kV).

## Results

### The serovars of *R. anatipestifer*

In this study, the genome data of *R. anatipestifer* involved a total of eleven serovars, including Serovar U1 (*n* = 10), Serovar 2 (*n* = 11), Serovar 3 (*n* = 1), Serovar 4 (*n* = 1), Serovar 5 (*n* = 1), Serovar 6 (*n* = 3), Serovar 9 (*n* = 1), Serovar 11 (*n* = 1), Serovar 8 (*n* = 5), Serovar U2 (*n* = 10), and Serovar 12 (*n* = 1), which were determined by slide agglutination or from references. All strains and their information are shown in Supplementary Table 1.

### The gene cluster associated with serovar phenotype of *R. anatipestifer*

**Fig. 1:**
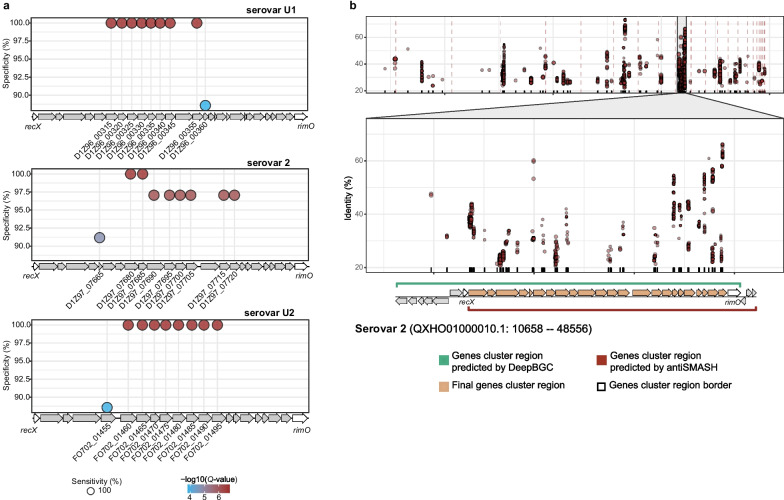
Location of genes significantly associated with serovars. **a** And the specificity and the sensitivity of genes significantly associated with serovars U1, 2, and U2. The size of the shape indicates sensitivity; colour indicates negative log of adjusted *P*-value. **b** Gene cluster location and boundary determination. The dot plot represents the hits of genes related to CPS on the genome, and the size of the dot indicates the coverage length. Interval markers on gene clusters indicate the BGC regions predicted by DeepBGC and antiSMASH

To screen for loci associated with serovars, the GWAS was performed with Scoary on the serovars containing more than 10 strains (serovars U1, 2, U2). According to the Pan-GWAS filtering threshold, we obtained a total of 27 target genes, and each serovar harbors 9 associated genes (Fig. 1a, Supplementary Table 3). Next, we mapped these genes to the corresponding genome and found that these genes were close to each other and formed a gene cluster. Interestingly, according to the BGCs results predicted by antiSMASH, the gene clusters mentioned above were labelled as polysaccharide biosynthetic gene clusters. Furthermore, the presence of *wza* and *wzc* gene indicates that this gene cluster is responsible for the biosynthesis of CPS. (Supplementary Table 4). Based on these results, we speculate that the serovar-specific gene cluster was CPS biosynthesis gene cluster of *R. anatipestifer*.

**Fig. 2:**
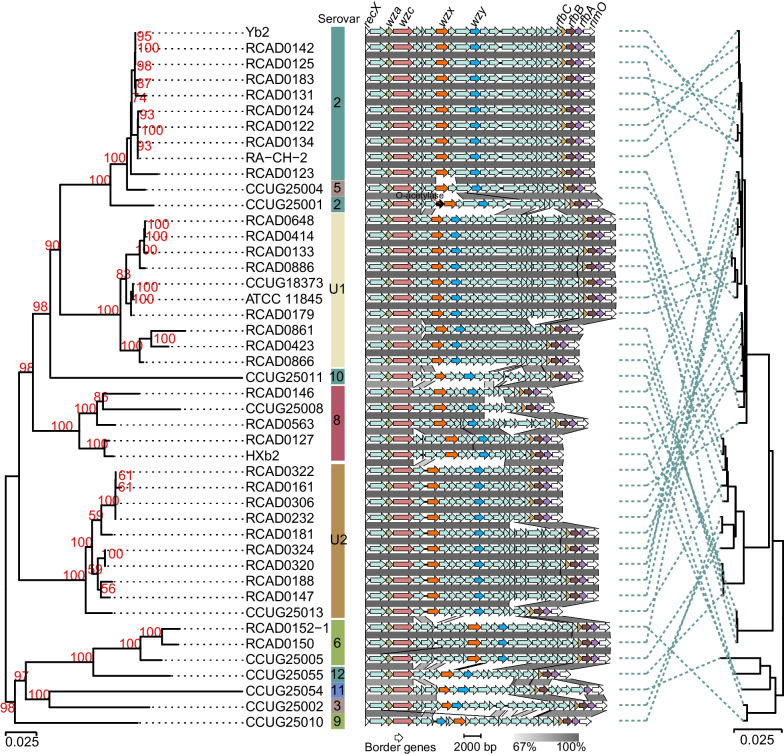
Comparison between CPS gene cluster phylogeny and core genome phylogeny. NJ phylogenetic tree and genetics structure of the CPS gene cluster are shown on the left, and the core genome phylogenetic tree is shown on the right. The same strain IDs are linked using dashed lines. Bootstrap values (greater than 50%) are indicated in red

We further compared the distribution of the gene cluster among different serovars, and the results showed that the position of the gene cluster was relatively conserved in the genome of *R. anatipestifer* (Fig. 2). In short, the gene region has conserved fragments of 4 and 5 genes at the beginning and end, respectively (Fig. 2).

To determine the boundaries of the CPS gene cluster, we focused on those locations with a high density of genes associated with CPS synthesis. The results show a distinct boundary in the region of the putative CPS gene cluster (Fig. 1b, Supplementary Fig. 2). Therefore, we speculate that that the CPS gene cluster of *R. anatipestifer* is located between the regulatory protein coding gene *recX* and the ribosomal protein S12 methylthiotransferase coding gene *rimO*, both of which are highly conserved in the genome of *R. anatipestifer* (Fig. 2).

### Inter- and intra-serovars comparison of CPS gene cluster

Based on the positional conservation of the CPS gene cluster, we extracted the CPS gene cluster sequences from serovar representative strains (Supplementary Table 5). The length of the gene clusters from 22.76 kb (serovar 8) to 30.18 kb (serovar U1), GC content between 32.55% (serovar 10) and 34.05% (serovar 2), which was significantly different from the genomic GC content (upper quartile: 34.98%, lower quartile: 34.82%, mean: 34.95%; paired t-test: *p*-value < 0.0001). We annotated the CPS gene clusters of the serovar representative strains, the results are shown in Supplementary Table 4. These gene clusters contain an average of 23 CDSs (ranging from 19 to 27). It is worth noting that all serovar CPS gene clusters contain *wza*, *wzc, wzx, wzy, rfbA, rfbB, and rfbC* genes. The presence of the set of *wzx* and *wzy* genes indicates that CPS synthesis in *R. anatipestifer* may be a Wzx/Wzy-related processing pathways (Supplementary Table 4, Supplementary Fig. 3).

Furthermore, a NJ phylogenetic tree based on the complete sequence of the CPS gene cluster and a synteny analysis of the CPS gene cluster (DNA sequence identity cut-off: 69%) are shown in Fig. 2. As expected, strains of the same serogroup have more similar CPS gene cluster structures to each other and clearly cluster together in the same phylogenetic clade. Except for a gene insertion event in CCUG25001, the genetic structure of the CPS gene cluster of all serovar 2 strains were highly similar (Fig. 2). According to the annotation results of Prokka and PGAP, the predicted function of the inserted gene is O-acetylase involved in peptidoglycan or LPS synthesis. Additionally, the cluster of serovar 5 and those of serovar 2 differed by only two genes (*wzx* and a glycosyltransferase coding gene). The gene clusters of serovar U1, 6, U2 strains have considerable similarity within the serogroup. Despite the relative diversity of the gene clusters of serovar 8 strains, their *wzx* and *wzy* are also identical. Comparative phylogenetic analysis revealed that the evolutionary trends of the core genome is inconsistent with CPS gene clusters (Fig. 2).

**Fig. 3:**
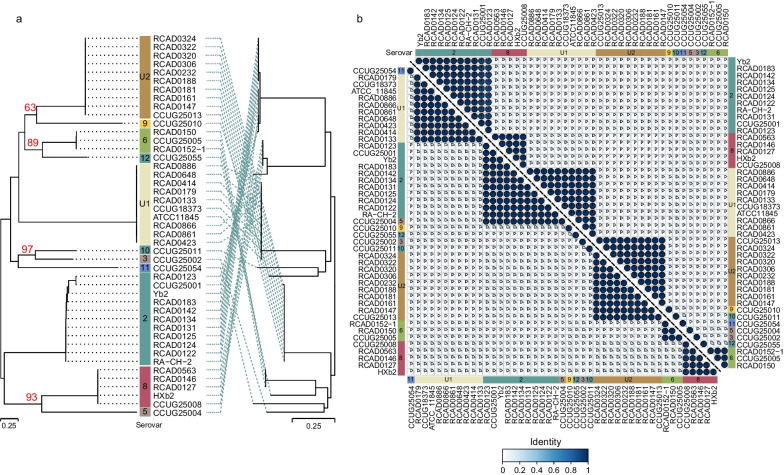
**a** Phylogenetic tree constructed by the neighbor joining method based on the Wzx (left) and Wzy (right) protein sequences. **b** Identity of Wzx (upper triangle) and Wzy (lower triangle) sequences

Next, we analysed the identity of the Wzx and Wzy sequences of all the strains by and constructed the NJ phylogenetic tree. Overall, Wzx and Wzy are serovar-specific, and much greater differences exist among the different serovars (Fig. 3a). Phylogenetic analysis of Wzx and Wzy, especially Wzx, clearly distinguishes the clades of different serovars. The minimum identity for the same serovars was 92.15% for Wzx and 95.14% for Wzy; the maximum identity for Wzx was 28.02% for different serovars and 26.50% for Wzy, except for serovars 5 with 2, which were 99.75% for Wzy (Fig. 3b).

### Conserved loci in other *Weeksellaceae* species

The synteny analysis of homologous gene clusters in *Weeksellaceae* indicated that the locus of the CPS gene cluster was conserved among closely related species (Supplementary Fig. 4 and 5). Specifically, the upstream gene arrangement (*recx*-*gdr*-*wza*-*wzc*) of *R. anatipestifer* CPS gene cluster was highly conserved. *Chryseobacterium* and *R. anatipestifer* were the same (*recx* and *rimO*) at the beginning and end of the region.

As expected, this locus is also conserved in *Elizabethkingia sp.* and *Chryseobacterium sp.* (Supplementary Fig. 4b and c). Furthermore, many glycosyltransferases related to polysaccharide synthesis are distributed in this region in both genera. It is worth mentioning that *rfbA, rfbB, and rfbC* (*Elizabethkingia sp.*), LPS export system ATP-binding protein gene (*lptB, Elizabethkingia sp.*), ligase gene (*Chryseobacterium sp.*), and oligosaccharide flippase gene (*Chryseobacterium sp.*) were also present in the conserved region, and they are usually involved in the synthesis of CPS and LPS.

Regarding the other two species of *Riemerella*: *Riemerella columbina* and *Riemerella columbipharyngis*, a similar gene cluster was found in *Riemerella columbina* DSM 16469 (Supplementary Fig. 4d). Furthermore, the genes encoding oligosaccharide repeat unit polymerase (Wzy) and oligosaccharide flippase (Wzx) were annotated in the cluster. However, compared with *R. anatipestifer*, the cluster region is significantly rearranged in *Riemerella columbina*. However, we could not detect similar genetic regions in *Riemerella columbipharyngis*.

### Identification and characterization of *R. anatipestifer* CH-2Δ*wzy*

The *wzy* of *R. anatipestifer* CH-2 was knocked out by allelic exchange, and the mutant CH-2Δ*wzy* was identified by PCR (Supplementary Fig. 1b). CH-2Δ*wzy* amplified the 16S rRNA fragment, Spec^R^ cassette fragment, and LSR fragment, but did not amplify the *wzy* fragment. All amplicons were confirmed by Sanger sequencing. After continuous culture for 30 generations, the genetic stability of the CH-2Δ*wzy* mutant was confirmed by the same PCR test (Supplementary Fig. 1c).

**Fig. 4:**
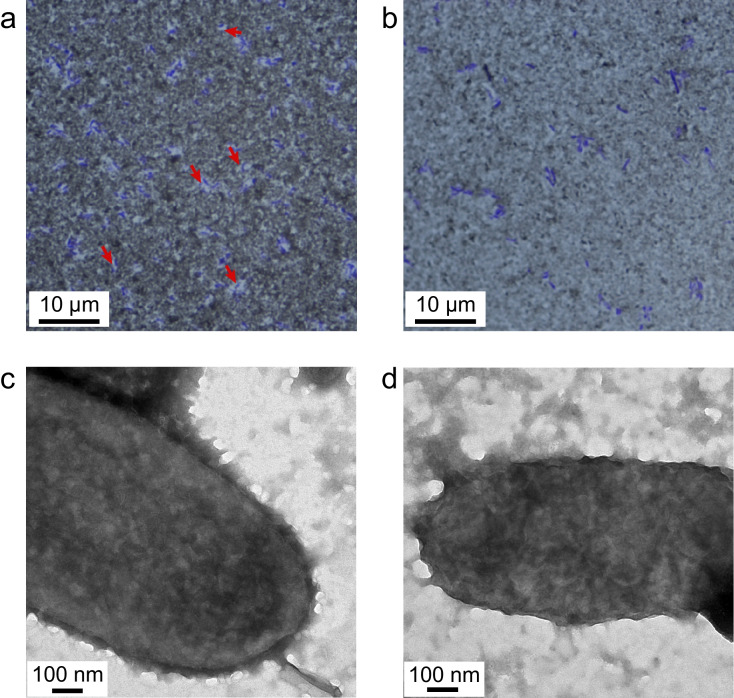
Capsule staining (**a**, **b**) and transmission electron microscope (**c**, **d**). The capsule of the wild-type strain CH-2 (**a**) and mutant strain CH-2Δ*wzy* (**b**) was stained and observed at 1000× magnification. The red arrows indicate the transparent capsule structures. The microstructure of wild-type strain CH-2 (**c**) and mutant strain CH-2Δ*wzy* (**d**) at 130000× magnification

The results of India ink staining showed that there was a white ring-like capsule structure around the wild-type strain CH-2 (Fig. 4a), while there was none around the mutant strain CH-2Δ*wzy* (Fig. 4b). The capsule structure observed by transmission electron microscopy indicated that there was a layer of furry substance on the surface of the wild-type strain (Fig. 4c), and the capsule of the mutant strain (Fig. 4d) was thinner than the wild-type strain. Furthermore, the antisera slide agglutination test indicated that CH-2Δ*wzy* could agglutinate with the antisera from multiple serovars (Supplementary Fig. 6).

## Discussion

Serotyping is an important method for characterizing *R. anatipestifer*, but corresponding molecular typing studies are still lacking. A recent study using time-of-flight mass spectrometry combined with machine learning algorithms has achieved recognition of serovars 1 and 2 [49]. Although the study was limited to a non-representative test set and did not indicate its corresponding molecular basis, its results suggest the feasibility of molecular serotyping of *R. anatipestifer*.

Pan-GWAS have been applied in various bacteria to explore the genetic basis of various phenotypes [22, 23, 50]. In the present study, we used Pan-GWAS to identify the genetic loci significantly associated with three prevalent serovars of *R. anatipestifer*. Further functional analysis of the loci suggested that these loci are responsible for the synthesis of CPS. The result is in agreement with our recent finding that the CPS of *R. anatipestifer* determines the serological specificity of the serovar 2 strain [19]. This is also consistent with previous studies in other species, suggesting that each serovar corresponds to a specific CPS synthesis gene cluster [9, 51, 52].

Based on the results of the association study between serovar and genome, we predicted and analysed the CPS gene cluster of *R. anatipestifer*. We observed that the CPS gene cluster exhibits a genetic structure with highly conserved regions at both ends and a diversified middle region. The structure is similar to that of *Klebsiella pneumoniae*, *E. coli*, and *Acinetobacter baumannii*, and *Vibrio parahaemolyticus*, with typical genes (i.e., *wza* and *wzc*) located in the start region of the gene cluster [10, 12, 53, 54]. Despite some minor differences, the phylogenetic relationships between gene clusters of the same serovar are closer together. There are non-essential gene differences in the CPS gene cluster of serovar 9 strains of *Streptococcus suis*, but these differences did not cause phenotype change [55]. Considering the significant differences in isolation time and geographical location between recent *R. anatipestifer* isolates and the serovar reference strains, these differences in the CPS gene cluster appear to be explainable. The CPS gene clusters of serovars 2 and 5 differ by only three genes, of which *wzx* is one of the key genes determining serological specificity. This phenomenon is also observed in other bacteria [10, 54, 56]. Notably, we observed phylogenetic inconsistency between the CPS gene clusters and the core genomes, which may be due to stronger selection pressure on capsule antigens [57]. The presence of *wzx* and *wzy* implies that the CPS of *R. anatipestifer* are processed via the Wzx/Wzy-dependent pathway [39]. *wzx* and *wzy* are widely used for capsule serotyping due to their excellent serovar specificity [12, 58], which has also been confirmed in our study. In *R. anatipestifer*, *wzx* can perfectly distinguish strains of different serovars, while *wzy* was slightly less effective as it cannot differentiate between serovar 2 and 5. Similar reports of two capsule serovars sharing the same *wzy* gene have been described in *Klebsiella spp* [56].

In this study, we also performed a conservative analysis of the CPS synthesis gene cluster of other *Weeksellaceae* species. It is noteworthy that a similar genetic locus is harbored in some species of *Chryseobacterium* and *Elizabethkingia*. To the best of our knowledge, there are no evidence-based reports of CPS synthetic gene clusters in *Chryseobacterium* and *Elizabethkingia*. Despite this limitation, we found several genes related to polysaccharide synthesis in these regions, such as *wbpA*, *wbpD*, *wbpE*, *lptB* and the ABC transporter ATP-binding protein gene [59, 60]. This is consistent with the previously putative capsular polysaccharide synthesis gene cluster of *Elizabethkingia* species [61]. Therefore, for some species of *Chryseobacterium* and *Elizabethkingia*, the above mentioned genomic region may also be the locus of the CPS synthesis gene cluster.

Furthermore, *wzy* gene (*G148_RS04365*) was deleted from *R. anatipestifer* CH-2. The absence of the capsule suggests that the *wzy* gene plays a crucial role in capsule synthesis, and previous study have shown that inactivation of *wza* in the CPS gene cluster also leads to the same phenotype [16]. Another study showed that knocking out the *AS87_04050* gene (coding Vi polysaccharide biosynthesis protein) from the CPS locus of *R. anatipestifer* Yb2 can alter serological characteristics, and the mutant strains exhibit a rough morphology [62], which is a typical feature of capsule loss [63]. And in the present study, the mutant strain CH-2Δ*wzy* could agglutinate with antisera from multiple serovar. Similarly, deletion of the *M949_1603* gene (coding glycosyltransferase family 2 protein) in *R. anatipestifer* CH-1 results in cross-reactivity [15]. And we have confirmed that *M949_1603* gene is also located in the CPS gene cluster region. One possible explanation for the phenomenon of cross-reactivity is that the absence of capsule leads to the exposure of highly conserved epitopes. These results may indicate that the CPS of *R. anatipestifer* is the major antigenic component related to serological characteristics.

## Conclusion

In this work, we revealed that association between the putative CPS gene cluster and the serovar types of *R. anatipestifer* through a genome-wide association studies. The CPS synthesis gene cluster of *R. anatipestifer* is serovar-specific. Moreover, the inactivation of the *wzy* gene results in defective capsule phenotype and cross-agglutination. This study provides new insights into the molecular basis of serotyping in *R. anatipestifer* and provides ideas for the development of molecular serotyping methods.

### Supplementary Information

**Additional file 1: Supplementary Table 1. **The strains and the published genome data used in this study. **Supplementary Table 2.** Information of 450 known capsular polysaccharide synthesis gene cluster from the NCBI. **Supplementary Table 3.** The specificity and the sensitivity of genes significantly associated with serovar U1, 2, and U2. **Supplementary Table 4.** Functional prediction of the gene in capsular polysaccharide synthesis gene cluster of serovar representative strain. **Supplementary Table 5.** Location, length and GC content of capsular polysaccharide synthesis gene cluster of serovar representative strain.

**Additional file 2: Supplementary Figure 1. **a) Construction of R. anatipestifer wzy mutant strain CH-2Δwzy. b) Identification of R. anatipestifer CH-2Δwzy. Lane M: DL2000 DNA Marker; Lanes 1-3: 16S rRNA F and 16S rRNA R, which amplify a 960 bp fragment from R. anatipestifer 16S rRNA. Order: Wild-type(CH-2), mutant(CH-2Δwzy), and negative control (distilled water); Lanes 4-6: Spec F and Spec R, which amplify a 1180 bp fragment from the SpecR cassette. Order: Positive control (pYES1 new), mutant (CH-2Δwzy), and negative control (distilled water); Lanes 7-9: wzy F and wzy R, which amplify an 886 bp fragment from the wzy gene. Order: Wild-type(CH-2), mutant (CH-2Δwzy), and negative control (distilled water); Lanes 10-11: LSR F and LSR R, which amplify a 1199 bp fragment from the SpecR cassette, indicating that it was inserted in the correct position in the R. anatipestifer CH-2 genome. Order: Mutant (CH-2Δwzy), Negative control (distilled water). c) Identification of R. anatipestifer CH-2Δwzy after continuous culture for 30 generations. Lane M1: DL15000 DNA Marker; Lane M2: DL2000 DNA Marker. The rest of the lanes are identical to (b). **Supplementary Figure 2.** Gene cluster location and boundary determination of serovar U1 and serovar U2. The dot plot represents the hits of genes related to CPS on the genome, and the size of the dot indicates the coverage length. Interval markers on gene clusters indicate the BGC regions predicted by DeepBGC and antiSMASH. **Supplementary Figure 3.** The prediction of transmembrane helices in amino acid sequences encoded by wzx and wzy. **Supplementary Figure 4.** Conserved loci in other Weeksellaceae species. a) The genetic locus of the CPS biosynthesis gene cluster in R. anatipestifer is conserved among the closest species. b) Conserved structure in multiple Elizabethkingia species. c) Conserved structure in multiple Chryseobacterium species. d) Comparison of the CPS gene cluster between R. anatipestifer (CH-2) and R. columbina (DSM 16469). **Supplementary Figure 5.** 16S rDNA NJ phylogenetic tree of closely related species (representative genome from NCBI) for R. anatipestifer. 16S rDNA nucleotide sequence alignment was performed using MAFFT and tree was reconstructed by MEGA X with default parameters and 1000 bootstrap replicates. **Supplementary Figure 6.** Agglutination test of R. anatipestifer mutant CH-2Δwzy. The Mutants are capable of reacting to multiple antisera. And the details of the reaction differed from the wild type.

## Data Availability

All genomic data generated or analysed in this study are archived in the NCBI Genome Database (https://www.ncbi.nlm.nih.gov/assembly/) and accession numbers can be found in Supplementary Table 1.
