# Supplementary material for: Genome-wide association study reveals serovar-associated genetic loci in Riemerella anatipestifer
Source: BMC Genomics. 2024 Jan 13;25:57. doi: 10.1186/s12864-024-09988-4 (PMC10787497; doi:10.1186/s12864-024-09988-4)
Supplement: Supplementary file 2 — Additional file 2: Supplementary Figure 1. a) Construction of R. anatipestifer wzy mutant strain CH-2Δwzy. b) Identification of R. anatipestifer CH-2Δwzy. Lane M: DL2000 DNA Marker; Lanes 1-3: 16S rRNA F and 16S rRNA R, which amplify a 960 bp fragment from R. anatipestifer 16S rRNA. Order: Wild-type(CH-2), mutant(CH-2Δwzy), and negative control (distilled water); Lanes 4-6: Spec F and Spec R, which amplify a 1180 bp fragment from the SpecR cassette. Order: Positive control (pYES1 new), mutant (CH-2Δwzy), and negative control (distilled water); Lanes 7-9: wzy F and wzy R, which amplify an 886 bp fragment from the wzy gene. Order: Wild-type(CH-2), mutant (CH-2Δwzy), and negative control (distilled water); Lanes 10-11: LSR F and LSR R, which amplify a 1199 bp fragment from the SpecR cassette, indicating that it was inserted in the correct position in the R. anatipestifer CH-2 genome. Order: Mutant (CH-2Δwzy), Negative control (distilled water). c) Identification of R. anatipestifer CH-2Δwzy after continuous culture for 30 generations. Lane M1: DL15000 DNA Marker; Lane M2: DL2000 DNA Marker. The rest of the lanes are identical to (b). Supplementary Figure 2. Gene cluster location and boundary determination of serovar U1 and serovar U2. The dot plot represents the hits of genes related to CPS on the genome, and the size of the dot indicates the coverage length. Interval markers on gene clusters indicate the BGC regions predicted by DeepBGC and antiSMASH. Supplementary Figure 3. The prediction of transmembrane helices in amino acid sequences encoded by wzx and wzy. Supplementary Figure 4. Conserved loci in other Weeksellaceae species. a) The genetic locus of the CPS biosynthesis gene cluster in R. anatipestifer is conserved among the closest species. b) Conserved structure in multiple Elizabethkingia species. c) Conserved structure in multiple Chryseobacterium species. d) Comparison of the CPS gene cluster between R. anatipestifer (CH-2) and R. columbina (DSM 16469). Supp [file 12864_2024_9988_MOESM2_ESM.docx]

## Supplementary Figure


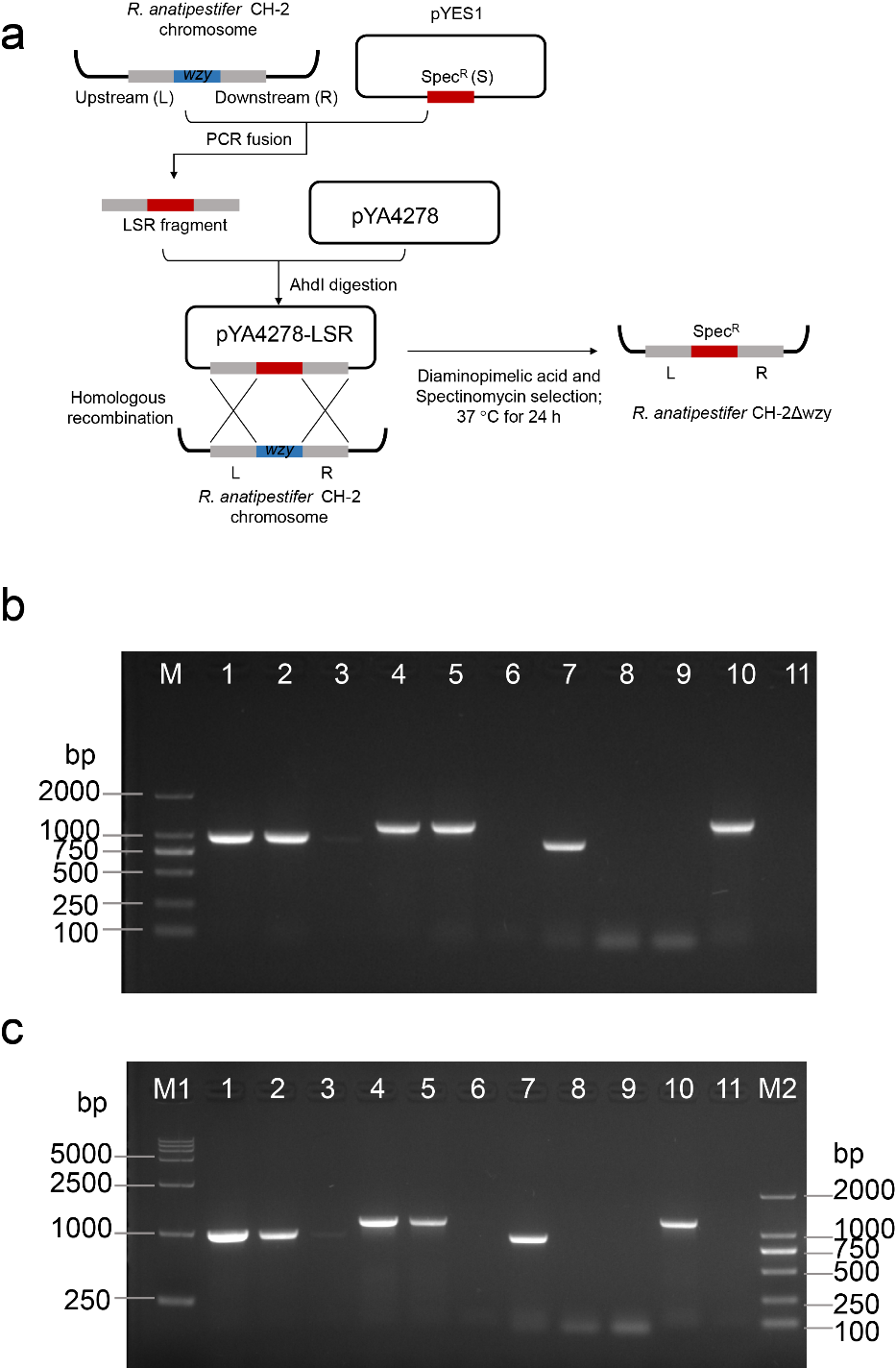


**Supplementary Figure 1. a)** Construction of *R. anatipestifer* *wzy* mutant strain CH-2Δ*wzy*.

**b)** Identification of *R. anatipestifer* CH-2Δwzy. Lane M: DL2000 DNA Marker; Lanes 1-3: 16S rRNA F and 16S rRNA R, which amplify a 960 bp fragment from *R. anatipestifer* 16S rRNA. Order: Wild-type(CH-2), mutant(CH-2Δ*wzy*), and negative control (distilled water); Lanes 4-6: Spec F and Spec R, which amplify a 1180 bp fragment from the SpecR cassette. Order: Positive control (pYES1 new), mutant (CH-2Δwzy), and negative control (distilled water); Lanes 7-9: wzy F and wzy R, which amplify an 886 bp fragment from the wzy gene. Order: Wild-type(CH-2), mutant (CH-2Δ*wzy*), and negative control (distilled water); Lanes 10-11: LSR F and LSR R, which amplify a 1199 bp fragment from the SpecR cassette, indicating that it was inserted in the correct position in the *R. anatipestifer* CH-2 genome. Order: Mutant (CH-2Δ*wzy*), Negative control (distilled water).

**c)** Identification of *R. anatipestifer* CH-2Δwzy after continuous culture for 30 generations. Lane M1: DL15000 DNA Marker; Lane M2: DL2000 DNA Marker. The rest of the lanes are identical to (b).


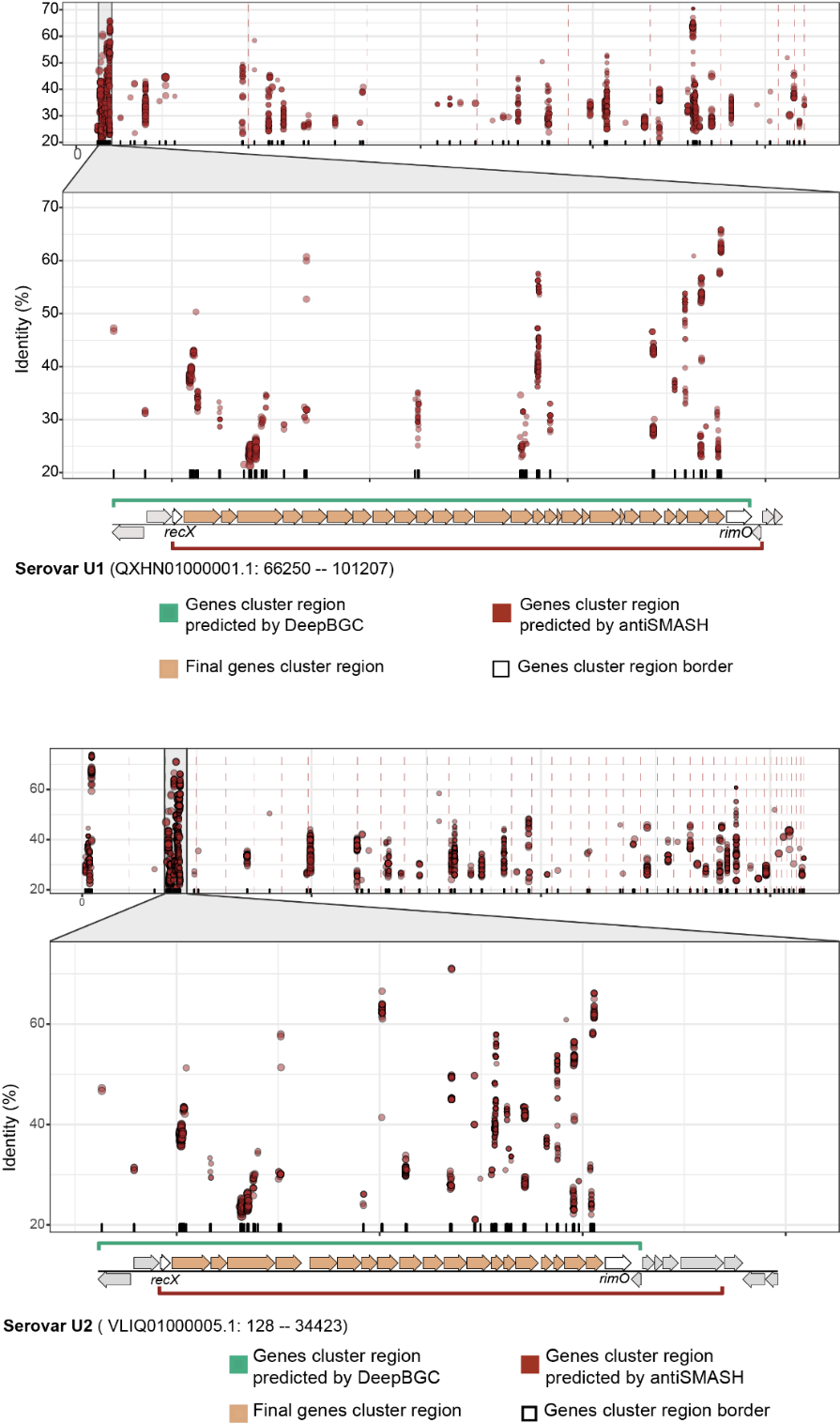


**Supplementary Figure 2.** Gene cluster location and boundary determination of serovar U1 and serovar U2. The dot plot represents the hits of genes related to CPS on the genome, and the size of the dot indicates the coverage length. Interval markers on gene clusters indicate the BGC regions predicted by DeepBGC and antiSMASH.


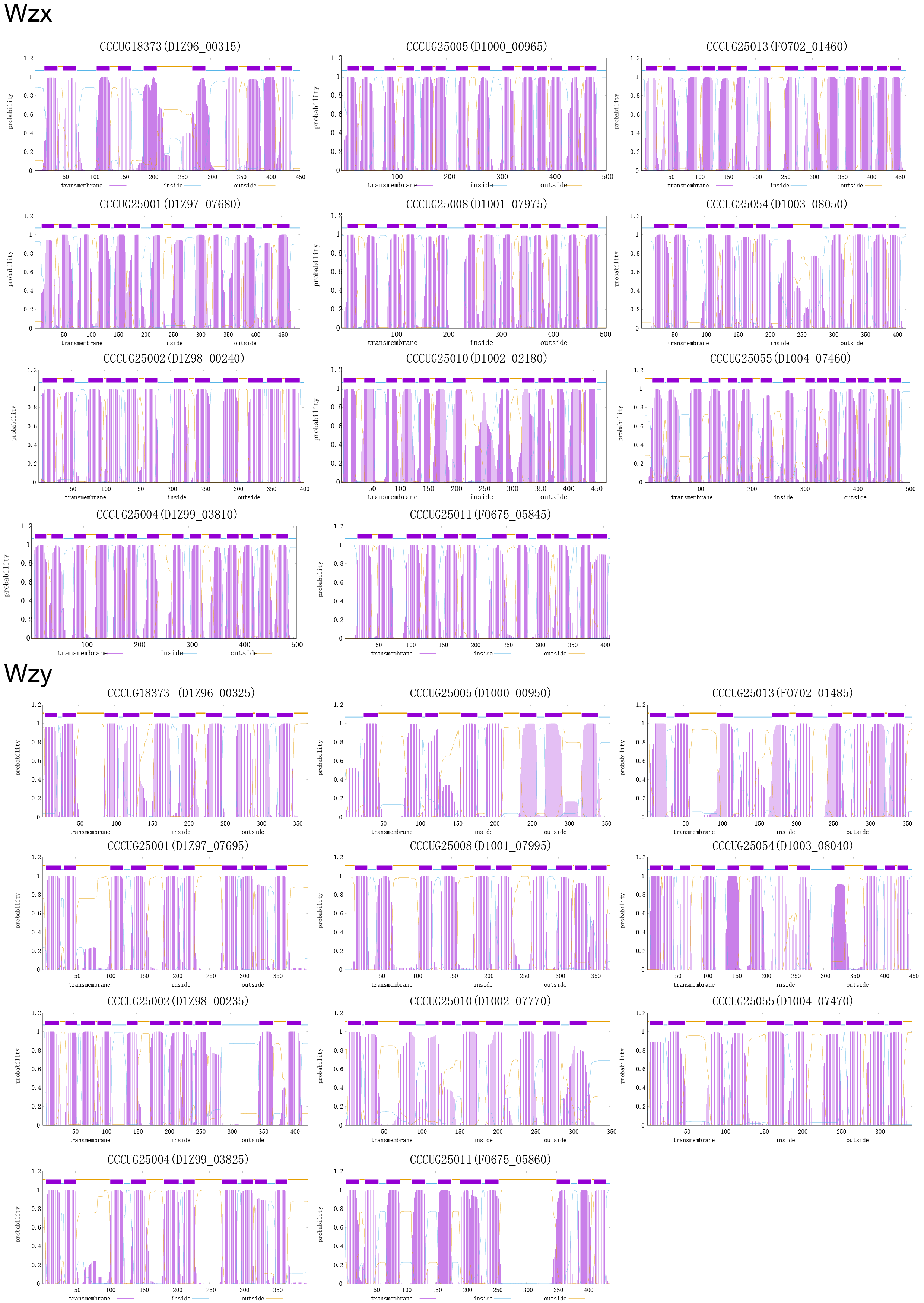


**Supplementary Figure 3.** The prediction of transmembrane helices in amino acid sequences encoded by *wzx* and *wzy*.


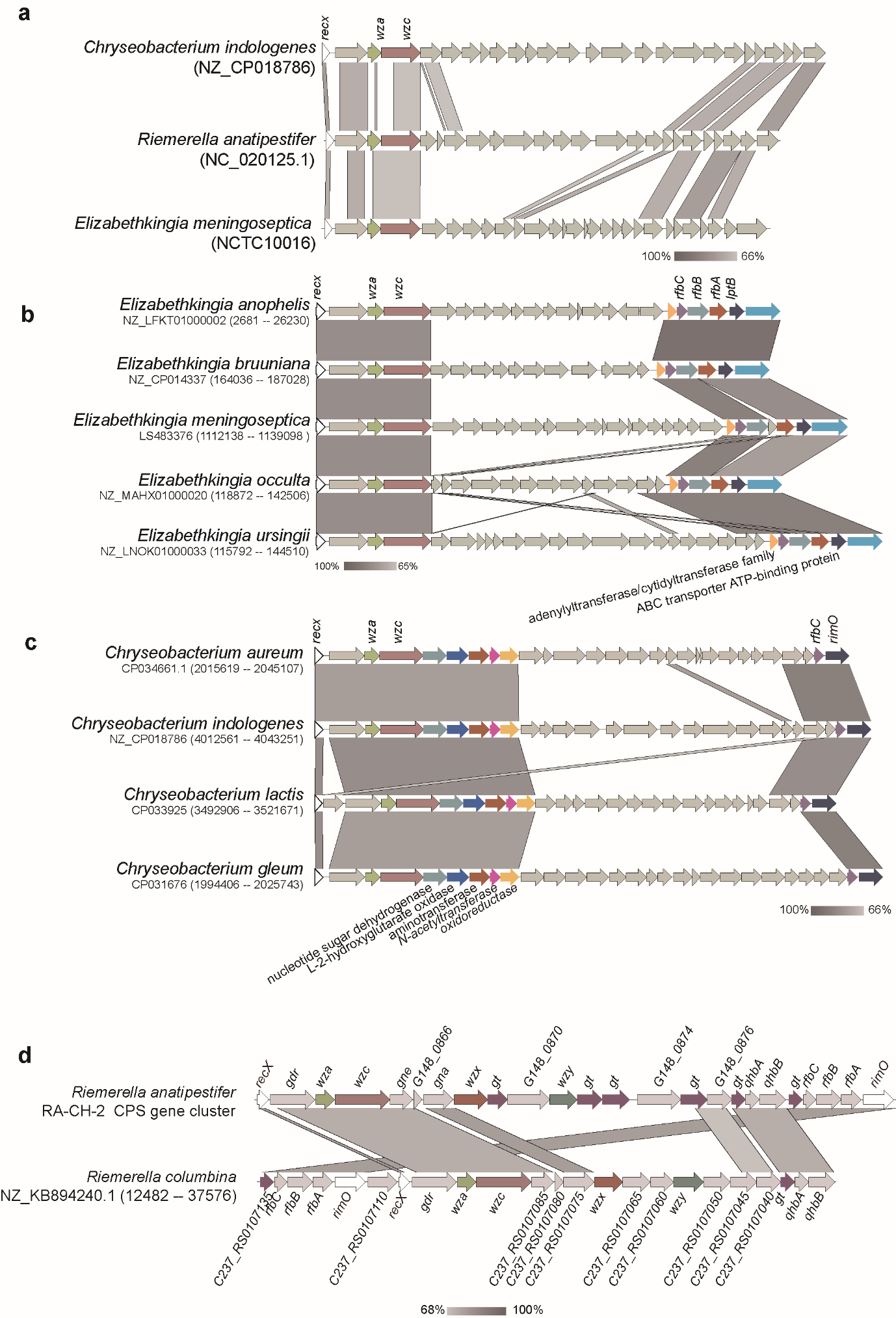


**Supplementary Figure 4.** Conserved loci in other *Weeksellaceae* species. a) The genetic locus of the CPS biosynthesis gene cluster in *R. anatipestifer* is conserved among the closest species. b) Conserved structure in multiple *Elizabethkingia* species. c) Conserved structure in multiple *Chryseobacterium* species. d) Comparison of the CPS gene cluster between *R. anatipestifer* (CH-2) and *R. columbina* (DSM 16469)


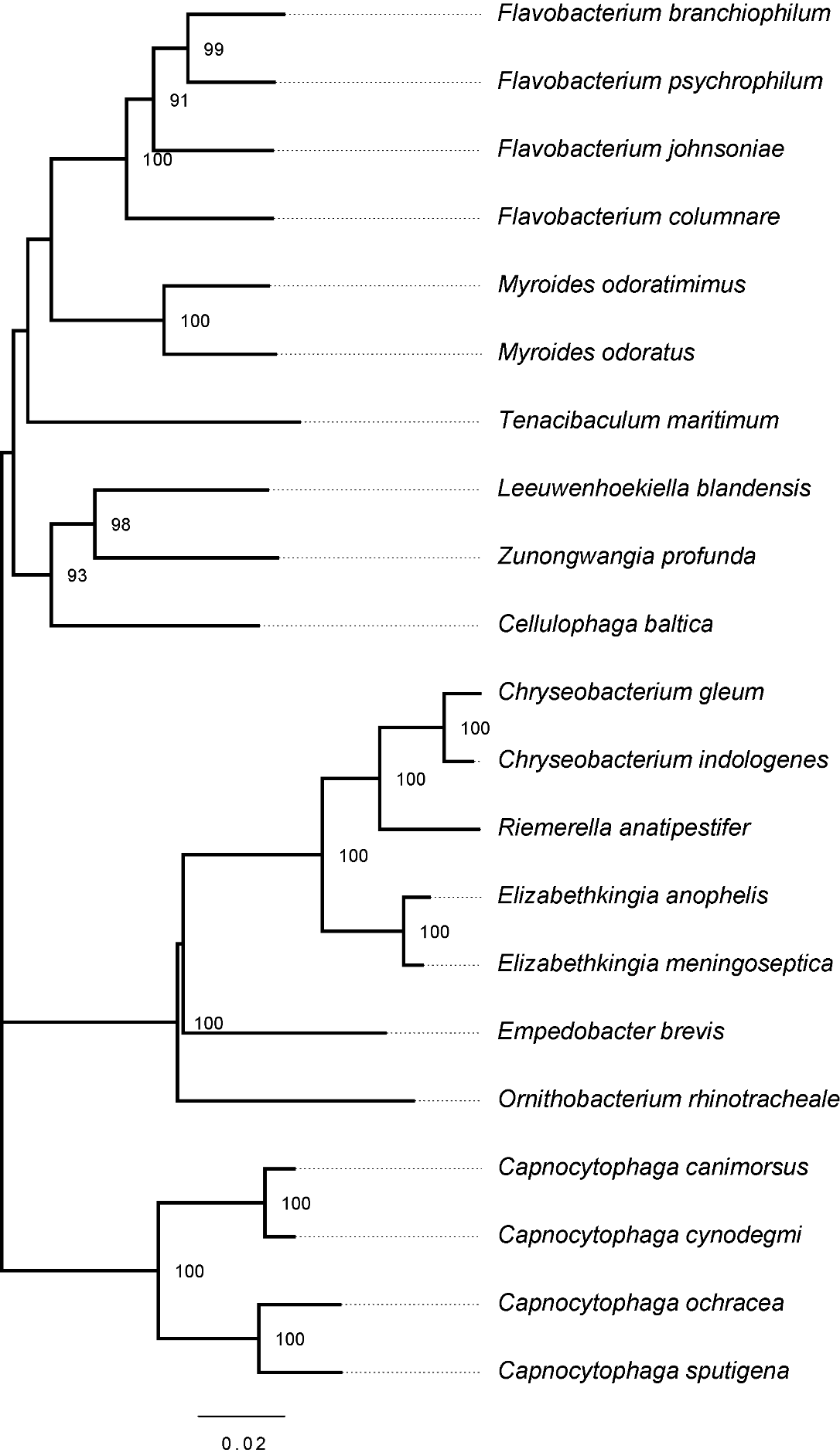


**Supplementary Figure 5** 16S rDNA NJ phylogenetic tree of closely related species (representative genome from NCBI) for *R. anatipestifer*. 16S rDNA nucleotide sequence alignment was performed using MAFFT and tree was reconstructed by MEGA X with default parameters and 1000 bootstrap replicates.


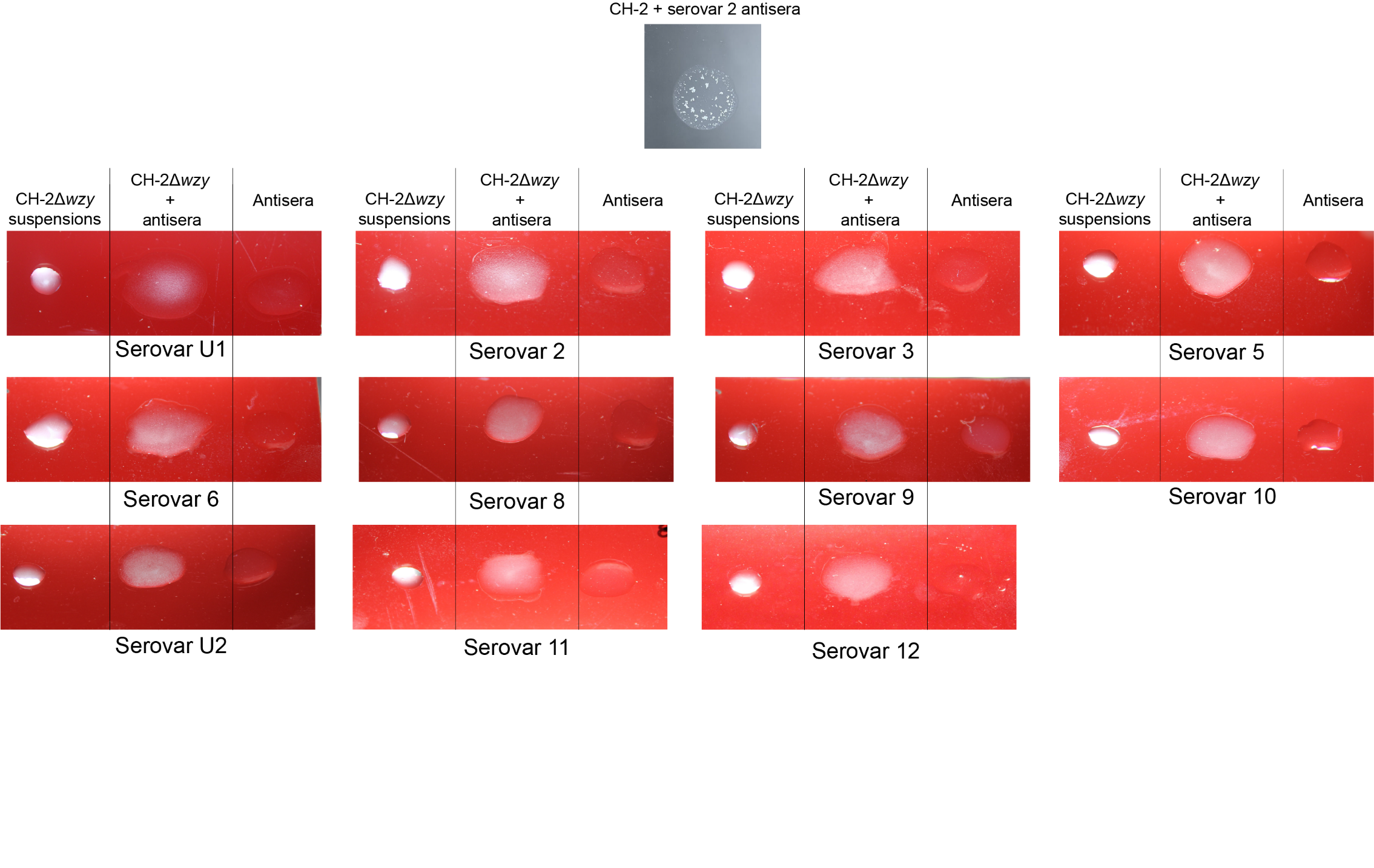


**Supplementary Figure 6** Agglutination test of *R. anatipestifer* mutant CH-2Δ*wzy.* The Mutants are capable of reacting to multiple antisera. And the details of the reaction differed from the wild type.
